# Is it safe to resume large scale in-person medical meetings?

**DOI:** 10.1590/S1677-5538.IBJU.2022.0210

**Published:** 2022-04-30

**Authors:** Cristiano M. Gomes, Julia Duarte de Souza, Karin M. J. Anzolch, João Victor T. Henriques, Lucas Nogueira, Eduardo Pimentel, Roni de C. Fernandes, Alfredo F. Canalini, José de Bessa

**Affiliations:** 1 Faculdade de Medicina da Universidade de São Paulo Divisão de Clínica Urológica São Paulo Brasil Divisão de Clínica Urológica, Faculdade de Medicina da Universidade de São Paulo, São Paulo, Brasil; 2 Hospital Moinhos de Vento Departamento de Urologia Porto Alegre RS Brasil Departamento de Urologia, Hospital Moinhos de Vento, Porto Alegre, RS, Brasil; 3 Universidade Federal de Minas Gerais Hospital das Clínicas Departamento de Urologia Belo Horizonte MG Brasil Departamento de Urologia do Hospital das Clínicas, Universidade Federal de Minas Gerais – UFMG, Belo Horizonte, MG, Brasil; 4 Hospital de Base do Distrito Federal Departamento de Urologia Brasília DF Brasil Departamento de Urologia, Hospital de Base do Distrito Federal, Brasília, DF, Brasil; 5 Santa Casa de São Paulo Faculdade de Medicina São Paulo SP Brasil Faculdade de Medicina, Santa Casa de São Paulo, São Paulo, SP, Brasil; 6 Universidade do Rio de Janeiro Departamento de Urologia Rio de Janeiro RJ Brasil Departamento de Urologia, Universidade do Rio de Janeiro - UERJ, Rio de Janeiro, Rio de Janeiro, RJ, Brasil; 7 Universidade de Feira de Santana Departamento de Cirurgia Feira de Santana BA Brasil Departamento de Cirurgia, Universidade de Feira de Santana, Feira de Santana, BA, Brasil

## INTRODUCTION

Participating in medical meetings is important for medical and other healthcare professionals with different backgrounds. The brightest minds share their expertise and present cutting-edge advancements in their field of knowledge [1]. The COVID-19 pandemic has had broad consequences for medical meetings worldwide. In support of public authorities in their effort to slow the spread of the disease, in-person medical meetings have been cancelled throughout the World since March/2020, or transformed in a virtual-only format, where the audience used online access. Most lectures, panel discussions and point-counter-point sessions are usually recorded in advance, through an online chat service, with no in-person interactions between speakers.

Although online meetings have served as a strong tool for medical education since the beginning of the pandemics, people are getting exhausted with this format. Being on a video call or teleconference requires more focus than face-to-face chat and lectures because it is harder to process non-verbal cues like facial expressions, the tone and pitch of the voice, and body language. Paying more attention to this drains a lot of energy. In addition, many of us are using video calls at work, family celebrations and interaction with friends. Everything seems to be happening in the same place, which further contributes to negative feelings for online meetings. Moreover, the online meeting is a reminder of the people, opportunities, and lifestyle that we have lost temporarily (2).

In-person meetings have been the most common and preferred format of medical conferences. They allow participants to interact with leaders and novices in the field, providing a unique networking experience that can foster future collaborations, help build a reputation, and create funding and career opportunities. In addition, browsing through the booths or presentations from vendors allows access to the latest technologies and tools in the field. However, social distancing is still effective throughout the World as we prepare this manuscript, and one cannot predict when in-person meetings will be safe again. Recent attempts to resume in-person medical meetings have been frustrated by the resurgence of COVID-19 due to the omicron variant. The Annual Congress of the European Association of Urology is one of the most important scientific meetings in the specialty and has been postponed from March/2022 to July/2022 due to the COVID-19 resurgence. Many other large meetings initially scheduled for the first semester of 2022 have been postponed, including the 52nd World Economic Forum Annual Meeting which was deferred from January to late May 2022.

In this study, we report our experience with a large medical meeting that took place in Brasilia, Brazil, in December/2021. We hypothesized that with most in-person participants being fully vaccinated and using proper precautions the number of new COVID-19 cases would be very low and not different from online participants.

## MATERIALS AND METHODS

### Meeting characteristics

The Brazilian Meeting of Urology is organized every other year by the Brazilian Society of Urology. It is considered the third-largest urological meeting globally, with a historical audience of approximately 4,000 attendees per event. The organization committee of the 38th meeting edition faced many challenges due to the global COVID pandemics. Initially scheduled for August 2021, it was postponed to October and eventually was held from 12 to 15 of December 2021. A hybrid format was employed with real-time broadcasting of all face-to-face activities to those who opted not to attend in person. The meeting had a duration of four days, with 10 daily hours of educational activities including plenary sessions with a total of 28 hours, 8 subspecialty workshops (10 hours duration each) and 44 educational courses (each with 2 to 4 hours duration).

### Characteristics of the venue

The meeting was held at the Brasilia International Convention Center (CCIB), a venue with 65,000 m2 of built area with closed air conditioning system. Only two of the three floors of the venue were used, including the 3rd floor with an area of 12,000 m2 that includes lobby entrance, main plenary, exhibition area, VIP room, restaurant and living rooms and the 1st floor with the same area, containing the rooms where the courses were held.

### Safety protocols

**Figures 1:**
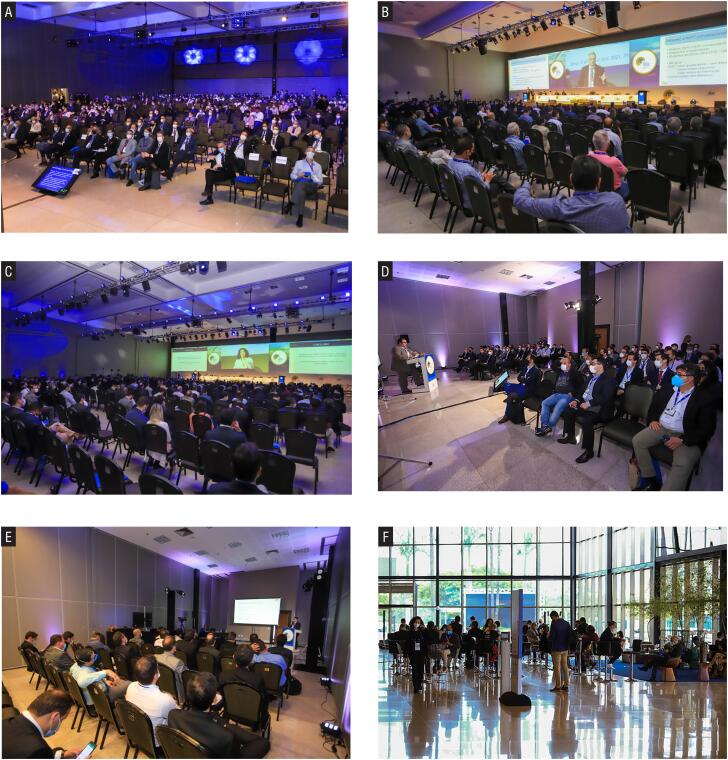
(A-F) - Measures were used to restrict participants per room, aiming to reach no more than 50% of the maximum room capacity.

Participants were not required to present proof of vaccination status nor test for SARS-CoV-2 before the event. The use of facemasks was mandatory in the venue except for the eating areas. Measures were used to restrict participants per room, aiming to reach no more than 50% of the maximum room capacity (Figures 1 A-F). On the first day of the meeting, every participant received a bottle of 70% alcohol gel and facemask in the meeting kit.

### Online survey

This study was conducted as an electronic cross-sectional survey sent by e-mail to all registered urologists who provided their e-mail address. There were no incentives for completion. The first e-mail inviting to participate was sent on January 10th/2022; four additional invitations were sent on January 12, 14, 17 and 21/2022. Data collection was closed on January 22/2022. A total of 1077 urologists who attended the meeting in-person were invited to participate in the study. Online participants (n= 781) served as a control group and were invited to complete the online survey at the same date, with the main purpose of identifying new cases of COVID-19. Since most online participants are urologists with similar lifestyle as the in-person participants we felt this would be an appropriate control group.

The invitation e-mail contained a link to a 10-question web-based survey. All questions were closed-ended, multiple choice. The survey included an assessment of previous COVID disease and vaccination status (Table-1).

**Table 1:** Baseline characteristics and new cases of COVID-19 after the meeting: Comparison between in-person and online participants.

	In-person participants (n = 309)	Online participants (n = 138)	P value
Age	42.0 (35.00-53.00)	43.5 (32.00-56.25)	0.634
Previous COVID-19	111 (35.92%)	39 (28.26%)	0.114
Vaccination status		**	0.989
No vaccination	2 (0.65%)	1 (0.72%)	
Incomplete	1 (0.32%)	1 (0.72%)	
Complete	55 (17.80%)	22 (15.94%)	
Complete + boost	251 (81.23%)	108 (78.26%)	
New onset COVID*	4 (1.29%)	6 (4.35%)	0.070

* Diagnosed within 15 days after the meeting

** 6 participants did not complete

### Risk of COVID-19 after the Meeting

Both in-person and online participants were asked whether they had been diagnosed with COVID-19 during the first 15 days after the end of the meeting.

The in-person participants were evaluated in terms of behaviors regarding the use of facemasks while at the meeting venue, time spent in the convention center during the meeting and concern regarding getting infected with SARS-CoV-2 during the meeting.

Informed consent was obtained from all participants.

### Data collection and Statistical analyses

Data were initially elaborated using Survey Monkey® software online. Data were expressed as medians and interquartile ranges, or absolute values and fractions. The Student t test was used to compare continuous variables while categorical variables were compared using the chi-square or Fisher’s exact tests. All tests were 2-sided with p <0.05 considered statistically significant and were performed using GraphPad Prism® version 9.03 for Windows.

## RESULTS

A total of 2608 subjects registered for the meeting including 1494 who participated in-person and 1114 who participated exclusively online. At the peak, 356 participants were online simultaneously. Respondents of the online survey included 309 (28.69%) of the in-person urologists and 138 (17.67%) of the online participants. The median age of onsite participants was 42 and the median age of the online participants was 43. The complete survey response rate was > 99% for both groups.

Onsite and online participants were comparable in terms of vaccination status against coronavirus. The vast majority in the two groups had received the complete vaccination scheme (99.03% vs 94.20%, in-person vs online participants, respectively; p= 0.989) and less than 1% in each group had not been vaccinated (Table-1). The groups were comparable in terms of age (p= 0.634), previous COVID-19 (p= 0.114) and vaccination status (p= 0.989). Participant’s characteristics (age, vaccination status, previous and new COVID diagnosis) are summarized in Table-1.

### Risk of COVID-19 after the Meeting

Four (1.29%) of the 309 in-person participants and six (4.35%) of the 138 online subjects reported being diagnosed with COVID within the fifteen days after the end of the meeting (p= 0.070).

Among the onsite attendants, 7 (2.2%) participants were at the meeting for one day, 33 (10.5%) for two days, 100 (31.8%) for three days and 174 (55.4%) for all four days. Regarding the use of facemasks, 217 (68.67%) onsite participants stated they wore a mask the whole time and briefly removed it during meals; 82 (25.95%) stated they wore a mask most of the time, but removed it for relatively long periods of time, on multiple occasions, for eating and other activities while 17 (5.38%) stated they removed their masks as much as they could.

Due to the small number of in-person participants who had COVID after the meeting, we could not evaluate whether vaccination status, mask-related behaviours or time spent at the convention center were associated with an increased risk of getting the disease.

As for the preoccupation of getting COVID-19 while attending the meeting, most participants (56.37%) stated they felt a little concerned, 39.17% were not concerned at all and 4.46% felt very concerned. Most (94.27%) considered the safety protocols at the convention center as adequate, while 4.78% found them deficient and 0.96% found them excessive. Only 33 (10.51%) onsite participants had attended other in-person medical event of similar or larger size during the pandemics.

## DISCUSSION

We have shown that participation in a large, indoor, medical meeting with nearly 1500 onsite participants and with four days duration held in December 2021 in Brasilia, Brazil, was not associated with increased SARS-CoV-2-infection risk. Four (1.29%) of the 309 in-person respondents became infected within 15 days of the meeting, compared with six (4.55%) of the138 online respondents, thereby confirming the absence of transmission risk. The groups were comparable in terms of history of previous COVID-19 and immunization status for the disease. Most onsite participants felt concerned about the risk of getting COVID-19 during the meeting, including 14 (4.46%) who were highly concerned and 177 (56.37%) who felt a little concerned.

Our finding of similar SARS-CoV-2-infection rates among in-person and online meeting attendees indicates that, in the context of high rates of immunization coverage against COVID-19 and low to medium circulation of SARS-CoV-2, the resumption of large medical meetings seems to be safe, as long as safety protocols are followed. Urologists in our control group have the same exposition risks in their daily activities as the in-person participants. In fact, it is reasonable to suppose that they are even more cautious and less exposed, since many may have opted not to attend in-person to minimize exposition.

From 12 to 15 of December 2021, when the meeting was held, the seven-day moving average of new daily cases in Brazil was 3,452 cases, which was close to the lowest rate of new cases in Brazil in 2021 (3). It was just before the outbreak of the omicron variant in Brazil, which was first detected in late November/2021 and increased the number of infections in the country to 8,000 cases/day and 100,000 cases/day 15 days and 30 days after the meeting, respectively. At the time of the meeting, 66% of Brazilians were fully vaccinated (4). Since urologists are healthcare workers and were prioritized by the government to receive vaccination, over 98% of both in-person and online meeting participants had been fully vaccinated, which was probably instrumental for the meeting to gather almost 1500 in-person attendants.

Few studies have evaluated the impact of participating in a major gathering since the outbreak of the COVID-19 pandemics. The SPRING study was the only randomized control trial where researchers evaluated the risk of attending a live indoor four-hour duration concert on May 29/2021, in Paris (5). Participants were healthy young men and women (18-45 years) that were evaluated for COVID-19 symptoms, recent case contact and had had a negative rapid antigen diagnostic test within 3 days before the concert. They were randomized to an experimental group (4451 attendees) or a control group (2227 non-attendees) and were tested for SARS-CoV-2 by RT-PCR on self-collected saliva 7 days post-gathering. Authors found no differences between the two groups in terms of positivity for SARS-CoV-2 seven days post-gathering (0.20% vs 0.15%, respectively for attendees and non-attendees). Less than 10% of the study population was fully vaccinated. They concluded that participation in a large, indoor, live gathering without physical distancing was not associated with increased SARS-CoV-2–transmission risk, provided a comprehensive preventive intervention was implemented. Other observational or small randomized controlled trials found similar results (6, 7). In common, these studies evaluated short duration events - of 3 to 5 hours duration - and all had stringent pre-event precautions. Our study, on the other hand, had no pre-event precaution and had a much longer duration, of 10 daily hours for up to 4 days.

To our knowledge, this is the first study reporting on the outcome of COVID-19 infections following attendance of a large medical meeting since the beginning of the pandemics. The event brought together around 1500 in-person participants for a meeting with four days of duration and showed a low risk of acquiring COVID-19 15 days after the meeting. The use of a control group composed of urologists that participated online gave us the opportunity to compare the rate of new cases among the onsite participants with that of a population of similar age and lifestyle that was not exposed to the risk of contagious associated with attending a large medical meeting.

Large indoor gatherings are considered high risk situations for Sars-CoV-2 transmission (8, 9), which justifies many people’s fear of attending such events. Among the meeting attendees, most were at least a little concerned about getting COVID, and for almost 90% this was the first large gathering they took part in since the beginning of the pandemic. Although the attendees were not required to present a negative COVID test, many other measures were taken as part of the preventive strategy, and the vast majority of participants found the implemented safety protocols to be adequate. Also, most reported adequate use of face masks. Combined with the high vaccination rate among participants, all these factors may have contributed to the safety of the meeting.

Our study does have significant limitations. First, the percentage of meeting attendants that participated in the survey was small and may not fully represent the whole population. Second, the diagnosis of COVID-19 was based on self-report, with no laboratory confirmation. In addition, it may not be appropriate to extrapolate our findings to other medical meetings in Brazil or other countries, since a number of conditions may vary, including the transmissibility of the circulating COVID-19 variants, the population’s vaccination status, the number and behavior of meeting participants and the size and characteristics of the venue where the meeting is held.

## CONCLUSIONS

In conclusion, our study showed that participation in a large, indoor, medical meeting with four days duration was not associated with increased SARS-CoV-2 infection risk, provided usual preventive measures were implemented. In the context of high rates of immunization coverage against COVID-19 and low to medium circulation of SARS-CoV-2, the resumption of large medical meetings seems to be safe. Our results may not be applied to the case of highly transmissible variants with shorter incubation period.
